# Post-Mortems

**Published:** 1891-01

**Authors:** 


POST-MORTEMS. What to Look For and How to Make Them. By A. H. Newth, London. Edited with numerous notes and additions by F. W. Owen, M. D., formerly Demonstrator of Anatomy, Detroit College of Medicine. Cloth, 12mo; post-paid, $1.00. The Illustrated Medical Journal Co., Publishers, Detroit, Mich.

This book is replete with information that every person interested in necroscopy should have at easy command. It has not been designed to take the place of large works upon pathology by its authors, but to present, in a tabulated way, with quick side-head references, all the important conditions of an organ met with post-mortemly, either in health or disease. To the country physician, who makes autopsies infrequently, it is especially valuable; also, to the medical student, who is occasionally in the "dead-house" of the hospital. It is the only brief work of the kind now at command. The American editor has made a great many examinations for court uses, and he has added numerous important notes to the text of the English author. Besides the ordinary conditions met with after death, there are chapters devoted to the post-mortem appearances seen in those poisoned, drowned, hanged, or cases of infanticide. It will thus be of great use in these classes of "suspected deaths." Full directions are also given for exposing the organs advantageously for their complete examination. The book will be sent, post-paid, upon receipt of price by its publishers.

